# Evaluation of CD44 Expression in Prostatic Adenocarcinoma: An Institutional Study

**DOI:** 10.7759/cureus.40510

**Published:** 2023-06-16

**Authors:** Priyanka V Damarasingu, Subhashish Das, Soumya MH, Sivaramakrishna Bodapati

**Affiliations:** 1 Pathology, Sri Devaraj Urs Academy of Higher Education and Research, Kolar, IND; 2 Urology, Sri Devaraj Urs Academy of Higher Education and Research, Kolar, IND

**Keywords:** targeted therapy, cancer, gleason’s grade, metastasis, trans urethral resection of prostate (turp)

## Abstract

Introduction: Prostate adenocarcinoma is the second-most common cause of cancer. Globally, many cancer-related deaths among men were noted due to prostate adenocarcinoma. CD44 plays a key role in mediating cell-to-cell and cell-to-matrix interaction, which further helps to maintain the integrity of tissue and also inhibits tumor metastasis.

Materials and methods: Cross-sectional study was done on chips from transurethral resections of the prostate (TURP) and prostatic core biopsy specimens. All specimens with clinically diagnosed and histopathologically confirmed prostatic adenocarcinoma were included in the study. Prostatic intraepithelial neoplasia (PIN), recurrent cases, and patients who had undergone radiotherapy/ chemotherapy prior to biopsy were excluded from the study. The sample size for the current study was 57 with an 8% prevalence value, 95% confidence interval, and 8% absolute error. Immunoreaction to CD44 antibody is membranous and was evaluated by calculating positively stained cell percentage and staining intensity. These two parameters were added to obtain a final score; a score of 0-3 was considered as negative, and a score of 4-6 was regarded as positive.

Results: A statistically significant difference was only found between Gleason grade (p<0.001), clinical staging (p<0.002), nodal metastasis (p<0.015), and distant metastasis (p<0.020) with CD44 positive expression. The rest of the parameters like PSA (p=0.642) and age (p=0.051) did not correlate with CD44-positive expression. Out of 29 cases with positive CD44 expression, 100% positivity was seen in Gleason’s grades 1, 2, and 3. This indicates that CD44 expression showed lesser positivity in poorly differentiated carcinoma. CD44 positivity was seen in 83.3% in the T2 stage. An inverse relationship between tumor staging and CD44 expression was observed with positive CD44 expression in lower tumor staging which implies loss of CD44 expression was associated with greater tumor aggressiveness. Lymph node metastasis cases showed more negative CD44 expression (59.5%) and the same was noted in patients without distant metastasis, that is in 61% of the subjects.

Conclusion: Cells tend to lose the ability of CD44 expression as they progress from well-differentiated adenocarcinoma to poorly differentiated adenocarcinoma. CD44 expression suggests that the tumor is in a well-differentiated and gland-forming state as compared to Gleason's grade. Loss of CD44 expression suggests tumor aggressiveness. Thus, the upregulation of CD44 expression can be considered as a potential target for targeted therapy. As many targeted and gene therapies are in clinical trials, large-scale multicentered studies are needed for a better understanding of the clinical course of the disease.

## Introduction

Prostate adenocarcinoma is the second-most common cause of cancer and cancer-related deaths among men worldwide [1]. Based on Gleason’s grading performed on hematoxylin and eosin sections, malignant potential can be determined. Alterations in the adhesion properties of neoplastic cells play a pivotal role in the transition of glandular to solid state. Among these adhesion molecules, CD44, a transmembrane glycoprotein, serves as the primary receptor of glycosaminoglycan hyaluronan, modulating migration/invasion during cancer progression. It plays an essential role in mediating cell-to-cell and cell-to-matrix interaction, promoting the maintenance of tissue integrity and inhibiting tumor metastasis [2-6]. Cancer cells undergoing an epithelial-to-mesenchymal transition (EMT) acquire stem cell-like properties and show increased CD44 expression with increased invasiveness and resistance to chemotherapy [7]. In the research done by Hassn et al., the expression of CD44 variants was associated with poor prognosis and metastasis in various cancers like colorectal, lung, ovarian, gastric, and breast [8]. However, limited studies were carried out regarding the correlation between Gleason’s grade and CD44-positive expression. Hence, this study was conducted to find out the association between CD44 expression and clinicopathological parameters (age, Gleason's grade, TNM staging of the disease, and prostate-specific antigen (PSA)).

## Materials and methods

Ethical clearance was obtained from the Institutional Ethics Committee (IEC) of our institute with IEC number SDUMC/KLR/IEC/474/2022-23. A cross-sectional study was done on transurethral resections of prostate (TURP) chips and prostatic core biopsy specimens received from the Department of Urology from 2016-2022 clinically diagnosed and histopathologically confirmed as prostatic adenocarcinoma by the Department of Pathology. All the clinical parameters (age, TNM staging of the disease, and PSA) were obtained from the archive of the Pathology Department. Hematoxylin and eosin slides were screened for histopathological type and Gleason’s grade of the tumors. Radiologic findings (USG, MRI, or CT findings) with respect to the disease stage and size of the lesion were noted.

Inclusion criteria

All prostatic adenocarcinoma which was clinically diagnosed and histopathologically confirmed either by prostate core biopsy or transurethral resection of the prostate (TURP) chips were included in the study.

Exclusion criteria

Prostatic intraepithelial neoplasia (PIN), patients with recurrence, and the subjects who had undergone radiotherapy/ chemotherapy were excluded from the study.

Sample size

The sample size was calculated using the formula Z_1-α2_ x p(1-p)/d2, where Z_1-α2_ = standard normal variant, p = expected proportion in population based on previous studies was taken as 8%, and d = absolute error was taken as 8%.

The sample size required for the cross-sectional study was calculated to be 57 for prostatic adenocarcinoma with a 95% confidence interval. Therefore, the sample size for the current study was 57 with an 8% prevalence value, 95% confidence interval, and 8% absolute error based on the study done by Thapliyal et al [2].

Method of IHC staining

**Figure 1:**
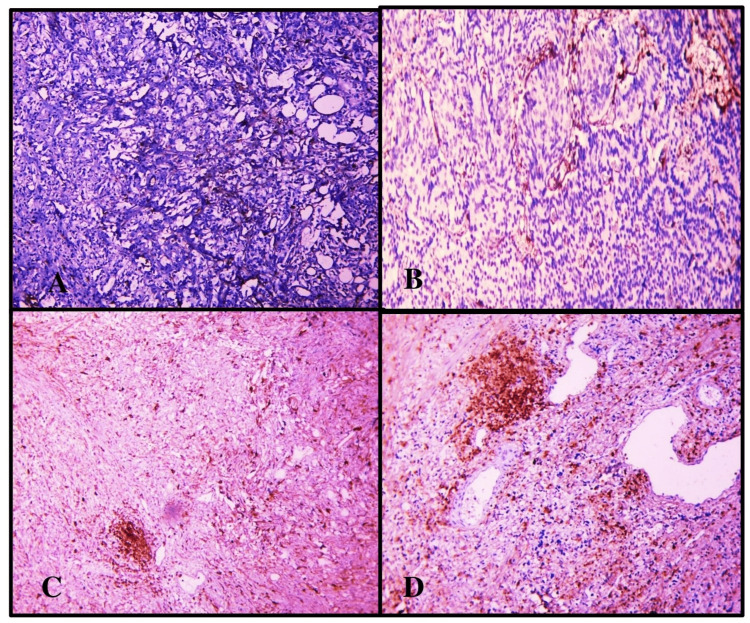
Panel A shows Score 0+0=0; Panel B shows Score 0+1=1; Panel C shows Score 1+1=2; Panel D shows Score 1+2=3. Panel A shows Score=0; here only 0-5% of tumor cells were showing CD44 expression with very weak intensity at 100X; B shows Score=1; 0-5% of tumor cells were expressing CD44 with weak intensity at 100X; Panel C shows Score=2, with 6-24% tumor cells expressing CD44 with weak intensity at 100X; Panel D shows Score=3; here, 6-24% of tumor cells were showing CD44 positivity with intermediate intensity at 100X.

**Figure 2:**
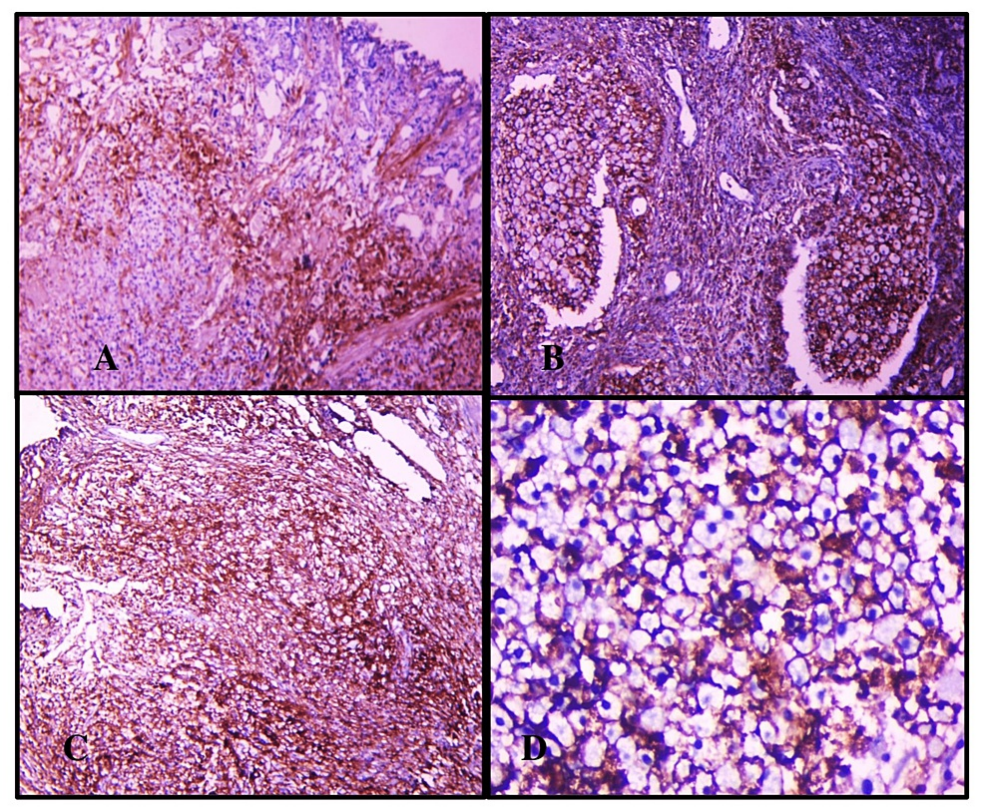
Panel A shows a score 2+2=4; Panel B shows a score 2+3=5; Panel C shows a score 3+3=6; Panel D shows a score 3+3=6. Panel A shows a score=4, which means 25-75% of the tumor cells were expressing CD44 with intermediate intensity at 100X; Panel B shows a score=5, with 25-75% of the tumor cells showing CD44 positivity with strong intensity at 100X; Panel C shows a score=6 with almost 76-100% tumor cells expressing CD44 with strong intensity at 100X; whereas Panel D shows a score=6 with CD44-positive expression at 400X.

Immunoreaction to CD44 antibodies (primary antibody-clone 156-3C11 and secondary antibody-horse radish peroxidase) was evaluated by calculating positively stained cell percentage and staining intensity and then added to obtain a final score. In the current study, we have utilized the indirect peroxidase blocking method for immunohistochemical evaluation with epithelial cells of the fallopian tube as positive control and fallopian tube stroma as negative control. Antigen retrieval was done by heat-induced epitope retrieval (HIER) was done utilizing a microwave. Standardization of CD44 expression was done with regard to antigen retrieval, the thickness of the section, and issues with primary/ secondary antibody fixation after undertaking necessary modifications. The expression of CD44 is membranous and was graded by taking two variables into account, as shown in Tables 1-2. A total score of 0-3 was considered as negative as shown in Figure 1 and a score of 4-6 was regarded as positive as shown in Figure 2 [2].

**Table 1:** Depicts the proportion of tumor cells positive for CD44-positive expression. A total score of 0-3 was considered as negative as shown in Figure 1 and a score of 4-6 was regarded as positive as shown in Figure 2 [2].

The proportion of cell percentage positive for CD44	
Score – 0	0-5%
Score – 1	6-24%
Score – 2	25-75%
Score – 3	76-100%

**Table 2:** Describes the intensity of staining of tumor cells for CD44 expression. A total score of 0-3 was considered as negative as shown in Figure 1 and a score of 4-6 was regarded as positive as shown in Figure 2 [2].

The intensity score staining for CD44	
Score - 0	Absent
Score - 1	Weak at 4x
Score - 2	Intermediate at 10x
Score - 3	Strong at 40x

Statistical analysis

SPSS software v.22 (IBM Corp., Armonk, NY) was used to analyze the data. Categorical data was represented in the form of frequencies and proportions. For qualitative data, we have used the chi-square test or Fischer's exact test (for 2x2 tables only) as a test of significance. Mean and standard deviation was used to represent continuous data. To identify the mean difference between two quantitative variables an independent t-test was used as a test of importance. The values were then presented in number (%) and mean ± SD. A p-value of < 0.05 was considered significant.

## Results

Out of 57 patients, 42 cases showed lymph node metastasis and 16 cases showed distant metastasis. Radiological assessment using MRI, CT, PET scans, and ultrasound was considered to assess metastasis.

In this study, the mean age among subjects without CD44 expression was 71.82±7.2 years and the mean age among the subjects with positive CD44 expression was 68.07±9.92 years, but no correlation was noted between the two.

Table 3 shows a correlation between PSA levels and CD44. PSA levels <30ng/ml (11 0f 13 cases) showed more CD44 positivity (84.6%) than PSA levels with 31-60ng/ml (seven of 16 cases); 61-90ng/ml (seven of 18 cases) and >90ng/ml (four of 10 cases) obtained less CD44 expression that was 43.8%, 38.9%, and 40% respectively.

**Table 3:** Comparison of association of CD44 with PSA levels among prostate adenocarcinoma cases The contents of this table show that as PSA levels increase, the expression of CD44 was decreased. Also, a rise in PSA levels reduced CD44 positivity. Among the cases with 61-90ng/ml of PSA, 11 out of 18 cases showed CD44-negative expression (61.1%), but there was no statistical significance (0.051) found between PSA levels and CD44. N=number of subjects; PSA=prostate specific antigen

PSA(ng/ml)	CD44 expression negative			CD44 expression positive		p-value
	Cases	N	%	N	%	0.051
<30	13	2	15.4%	11	84.6%	
31-60	16	9	56.3%	7	43.8%	
61-90	18	11	61.1%	7	38.9%	
>90	10	6	60.0%	4	40.0%	

Table 4 shows a comparison of CD44 expression with Gleason's grade and highlights that Gleason's grade 1 (six cases), Gleason's grade 2 (nine cases), and Gleason's grade 3 (six cases) showed 100% positivity for CD44. Loss of CD44 expression was noted as an increase in Gleason's grade as seen in Gleason’s grade 4 (eight of 10 cases) and Gleason’s grade 5 (20 of 26 cases) with 80% and 76.9% respectively.

**Table 4:** Comparison of association of CD44 with Gleason grade among prostate adenocarcinoma cases The contents of this table depict most of the cases expressing CD44 with Gleason’s grades 1, 2, and 3 showing a significant p-value of 0.001. This indicates that the lesser the Gleason’s grade more is the CD44 positivity, as 100% positivity was observed in Grades 1, 2, and 3. CD44 expression was reduced in Gleason’s grades 4 (80%) and 5 (76.9%). N=number of subjects

		CD44 expression negative		CD44 expression positive		p-value
Gleason’s Grade	Cases	N	%	N	%	0.001
Grade 1	6	0	0%	6	100.0%	
Grade 2	9	0	0%	9	100.0%	
Grade 3	6	0	0%	6	100.0%	
Grade 4	10	8	80.0%	2	20.0%	
Grade 5	26	20	76.9%	6	23.1%	

Table 5 depicts the correlation between T staging, nodal metastasis, distant metastasis, and CD44 expression. We found that the T2 stage (15 of 18 cases) showed increased expression of CD44 (83.3%). With the increase in the T staging, there was an increased loss of CD44 expression in T4 (13 of 17 cases) followed by T3 (12 of 22 cases) with 76.5% and 54.5% respectively. Among 15 subjects without lymph node metastasis, CD44-positive expression was seen in 12 patients which was around 80% which implies that there was greater expression in patients without lymph node metastasis. It also showed CD44 positivity in four cases with distant metastasis that was around 25% which further implies that CD44-positive expression was noted in well-differentiated tumors without lymph node metastasis and distant metastasis.

**Table 5:** Comparison of association of CD44 with clinical staging, nodal metastasis, and distant metastasis among prostate adenocarcinoma cases The contents of this table show a statistically significant difference between T staging, nodal metastasis, distant metastasis, and CD44 expression with p-values of 0.002, 0.015, and 0.020 respectively. Here, we can see the inverse relationship between T staging and CD44 expression with more expression in T2 (83.3%) than in T3 (45.5%) and T4 (23.5%). Lymph node metastasis cases showed more negative CD44 expression (59.5%) than lymph nodes without metastasis (20%). And the same was noted in patients without distant metastasis i.e. CD44-positive expression was noted in 61% and was seen only in 25% of cases with distant metastasis. N=number of subjects; T=tumor staging; N=nodal staging (lymph node); M=distant metastasis

Parameters		CD44 expression negative		CD44 expression positive		p-value
T staging	Cases	N	%	N	%	0.002
T1	0	0	0%	0	0%	
T2	18	3	16.7%	15	83.3%	
T3	22	12	54.5%	10	45.5%	
T4	17	13	76.5%	4	23.5%	
Nodal metastasis						
Absent	15	3	20.0%	12	80.0%	0.015
Present	42	25	59.5%	17	40.5%	
Distant metastasis						
Absent	41	16	39.0%	25	61.0%	0.020
Present	16	12	75.0%	4	25.0%	

A statistically significant difference was only found between Gleason grade (<0.001), clinical staging (p<0.002), nodal metastasis (p<0.015), and distant metastasis (p<0.020) with CD44-positive expression. The rest of the parameters such as PSA (p=0.642) and age (p=0.051) did not correlate with CD44-positive expression. The loss of CD44 expression was associated with greater tumor aggressiveness and positive CD44 expression was associated with a lower Gleason’s grade and vice versa. 

## Discussion

In prostate carcinoma, CD44 acts as a metastatic tumor suppressor gene [2]. Downregulation of CD44 at mRNA and protein during prostatic cancer progression results in high tumor grade, aneuploidy, and tumor metastasis [9-11]. RUNX-2 (Runt-related transcription factor 2) forms a complex with CD44-ICD (intra-cellular domain), thus activating the expression of metastasis-related genes [12]. CD44 variant isoforms like CD44v8-10 are usually expressed more in cancer stem cells and metastatic cells than CD44 standard isoforms [13]. Many studies showed that methylation of cytosine in the promoter region of CD44 leads to decreased CD44 expression in the human prostate [14-17].

**Table 6:** Highlights a comparison of the present study with other studies The contents of this table show that Gleason’s grading was indirectly proportional to CD44 expression with a p-value of <0.001, which was similar to a study done by Balci et al. but different from a study done by Thapliyal et al [2,3]. TNM staging was significant with p value<0.002, which was comparable to a study done by Carneiro et al. with a p-value <0.020 [18]. In contrast to other studies, our study did not show any correlation between age and PSA levels with CD44-positive expression [2,18]. T=tumor staging; N=nodal staging (lymph node); M=distant metastasis; PSA=prostate specific antigen

Parameters	Present study (p-value)	Carneiro et al., 2019 [18](p-value)	Balci et al., 2020 [3] (p-value)	Thapliyal et al., 2021 [2] (p-value)
Age	0.642	–	–	<0.024
Gleason’s Grade	<0.001	–	<0.001	0.278
T Staging	<0.002	<0.020	–	–
N Staging	<0.015		–	–
M Staging	<0.020		–	–
PSA Level	0.051	<0.034	–	<0.001

Table 6 shows a comparison of all parameters (age, Gleason's grading, TNM staging, and PSA levels) with the present study. In a study done by Thapliyal et al. on 63 cases, statistical significance with regards to age and CD44 expression was noted as they have included benign prostatic hyperplasia (BPH), prostatic intraepithelial neoplasia (PIN) cases along with prostate adenocarcinoma, unlike our study with only prostate adenocarcinoma cases [2]. Gleason’s grading and CD44-positive expression correlation were identical to the study done by Balci et al. in which they have taken BPH and prostate adenocarcinoma cases and concluded that there is a transition of loss of CD44 expression from BPH to prostate adenocarcinoma [3]. CD44 expression positivity was seen in Grades 1, 2, and 3 and most of the patients with grades 4 and 5 were negative, which corresponded to a study done by Balci et al. and Thapliyal et al [2,3]. Conversely, Thapliyal et al. did not find any statistical significance between Gleason's grading and CD44-positive expression probably due to the inclusion of an equal number of BPH (29) and prostate adenocarcinoma (30) cases in their study [2]. TNM staging was statistically significant in our study akin to Carnerio et al., but they have concluded that CD44-positive expression was noted in locally advanced cases (T3b) as they have taken TNM as a single parameter. On the contrary, in our study, we observed that CD44-positive expression was greater in T2 staging wherein we evaluated each parameter of TNM individually [18]. In our study, PSA levels did not correlate with CD44-positive expression, but conflicting results were noted in the studies done by Thapliyal et al. and Balci et al. as they have included BPH cases too [2,3]. 

Upregulation of CD44 expression by certain microRNAs can be utilized as a novel therapeutic agent against prostatic adenocarcinoma; if CD44 expression is found early, we can prevent the disease progression to advanced stages [3]. CD44 silencing in tumors can be the reason for basement membrane degradation and stromal-epithelial communication disruption [2]. CD44 can serve as an adverse prognostic marker among patients with cancer [19]. In a study done by Chen et al., silibinin, a drug that is under clinical trial, is known to inhibit CD44 and has the potential for clinical use [5].

Targeting CD44 and the Hippo-YAP pathway might be a potential treatment for docetaxel-resistant prostate adenocarcinoma, as docetaxel is a common drug used for metastatic prostatic carcinoma, migration, and invasion of docetaxel-resistant prostate carcinoma cells is promoted by CD44 via induction of Hippo-Yap signaling [20]. Studies have also shown that a combination of SB-3CT (matrix metallo proteinases=MMP2/9 inhibitor) and docetaxel was more effective in tumor growth inhibition in metastatic cases [21].

Overexpression of the NUMB gene (NUMB endocytic adaptor protein) in CD44-positive cancer stem cells inhibited invasiveness and clonal formation capabilities leading to tumor growth and metastasis inhibition [22]. According to a study by Iczkowski et al., CD44 can be a potential for gene therapy by stable re-expression [23].

To summarize, an inverse relationship was observed between CD44 expression with respect to Gleason’s grading and TNM staging and was found to be statistically significant (p-values <0.001, <0.002, <0.005, and <0.020) respectively. However, the exact pathophysiological mechanism of CD44 in the prostate still remains an enigma, so, further larger-scale studies and regular follow up are essential to obtain a better picture regarding the same. Contrary to other studies, PSA levels in the current study reflect the values in the elderly age group and their association with malignancy. This study highlights that there is a reciprocal relationship between CD44 expression and PSA levels predominantly in the elderly age group but was not statistically significant with a p-value of 0.642. However, more such studies are required for further validation.

Limitations of this study are that this is a unicentric study with a limited sample size and utilizes a solitary IHC marker without follow-up. 

## Conclusions

Cells tend to lose the ability of CD44 expression as they progress from well-differentiated adenocarcinoma to poorly differentiated adenocarcinoma. CD44 expression suggests that the tumor is in a well-differentiated and gland-forming state as compared to Gleason's grade and its loss suggests tumor aggressiveness. CD44-negative expression can be considered a hallmark of metastatic disease in prostate adenocarcinoma. CD44 positivity is associated with lower Gleason’s grades and tumor staging. Thus, the upregulation of CD44 expression can be considered a potential target for targeted therapy. As many targeted and gene therapies are in clinical trials, large-scale multicentered studies are needed for a better understanding of the clinical course of the disease.
